# Comparative Diagnostic Performance of Cardiac MRI and FDG-PET in Myocardial Inflammation: A Systematic Review and Meta-Analysis

**DOI:** 10.7759/cureus.100482

**Published:** 2025-12-31

**Authors:** Bareq S Al-Lami, Mustapha El Yaman, Abdulrahman O Saeed, Zhyar Y Mustafa, Ibrahim Chaheen, Sarah Aljuboori, Dema R Alasaly, Roz Wlya, Niga Alhayderi, Chro Bakr, Baqir Al-Lami, Yasir Al-Lami

**Affiliations:** 1 Radiology, Royal Sussex County Hospital, University Hospitals Sussex NHS Foundation trust, Brighton, GBR; 2 Conquest Hospital, East Sussex Healthcare NHS Trust, St. Leonards On Sea, GBR; 3 College of Medicine, Hawler Medical University, Erbil, IRQ; 4 General Medicine, International Federation of Medical Students' Associations (IFMSA) Kurdistan, Erbil, IRQ; 5 College of Medicine, Al-Mustansiriya University, Baghdad, IRQ

**Keywords:** cardiac mri, cardiac sarcoidosis, fdg-pet, myocardial inflammation, myocarditis

## Abstract

Cardiac inflammation, including myocarditis and cardiac sarcoidosis, poses significant risks and is a diagnostic challenge that is associated with heart failure, arrhythmias, and sudden cardiac death. Endomyocardial biopsy remains the reference standard but is limited by invasiveness, sampling error in patchy disease, and procedural risk. Noninvasive imaging, particularly cardiac magnetic resonance (CMR) and fluorodeoxyglucose F-18 positron emission tomography (18F-FDG-PET), is therefore central to diagnosis and follow-up. This systematic review and meta-analysis compared the diagnostic performance of CMR and FDG-PET, focusing on sensitivity, specificity, and clinical utility.

Following Preferred Reporting Items for Systematic Reviews and Meta-Analyses (PRISMA) 2020, we searched PubMed, MEDLINE, Embase, Scopus, and the Cochrane Library through October 2025. We included prospective and retrospective adult studies of suspected or confirmed cardiac inflammation undergoing CMR or FDG-PET. Two reviewers independently extracted data and assessed quality with QUADAS-2. Pooled sensitivity and specificity were estimated using a random-effects model, heterogeneity was assessed with the I² statistic, and hierarchical summary receiver-operating characteristic (HSROC) curves were used to examine threshold effects.

Twenty-six studies met the inclusion criteria (17 CMR, 9 FDG-PET). CMR showed consistent accuracy with a pooled sensitivity of 0.89 (95% CI 0.85-0.92) and a specificity of 0.87 (95% CI 0.83-0.90). FDG-PET had higher sensitivity, 0.93 (95% CI 0.89-0.96), but lower and more variable specificity, 0.71 (95% CI 0.62-0.79).

Both modalities are valuable and complementary. CMR’s high, reliable specificity and tissue characterization (edema, fibrosis) make it an appropriate first-line test without ionizing radiation. FDG-PET’s greater sensitivity suits the detection of active metabolic inflammation, particularly in sarcoidosis, when strict patient preparation limits false positives. These results support a stepwise approach: CMR first, with FDG-PET for equivocal cases or when assessment of active disease is needed.

## Introduction and background

Cardiac inflammation, encompassing conditions such as myocarditis and cardiac sarcoidosis, remains a challenging diagnostic and therapeutic problem in cardiovascular medicine. These disorders present with a broad clinical spectrum, ranging from nonspecific symptoms such as fatigue, dyspnea, and chest pain to severe manifestations including malignant arrhythmias, cardiogenic shock, and sudden cardiac death. Although myocarditis is the most commonly recognized entity in routine practice, other inflammatory conditions, particularly cardiac sarcoidosis, contribute substantially to morbidity and require distinct diagnostic and management strategies [1,2]. Viral infections remain the most frequent trigger for myocardial inflammation, with the SARS-CoV-2 pandemic further highlighting the clinical relevance of post-infectious cardiac involvement. Other recognized triggers include autoimmune disease, malignancy, and therapeutic exposures, most notably immune checkpoint inhibitors and vaccines [3-5].

Epidemiologic data suggest an increasing global burden of myocarditis, with cases rising from approximately 780,410 in 1990 to 1,265,770 in 2019, accompanied by a parallel increase in mortality. These estimates, however, derive from large administrative datasets and pertain specifically to myocarditis, and should not be extrapolated uncritically to other inflammatory cardiomyopathies such as cardiac sarcoidosis, which differ in case definitions, surveillance, and natural history [6-8]. Regardless of etiology, persistent or recurrent myocardial inflammation may lead to irreversible fibrotic remodeling, dilated cardiomyopathy, and progressive systolic dysfunction, underscoring the importance of accurate and timely diagnosis [9].

Endomyocardial biopsy (EMB) remains the histopathologic reference standard for confirming myocardial inflammation, but its role in routine practice and research is constrained by several limitations. EMB is invasive, carries procedural risk and cost, and is vulnerable to sampling error in diseases with patchy myocardial involvement. In addition, its availability and utilization vary widely across centers, depending on operator expertise and institutional practice patterns. As a result, EMB is often impractical as a universal comparator in diagnostic studies, increasing reliance on noninvasive imaging modalities for both diagnosis and longitudinal disease assessment [10,11].

Cardiac magnetic resonance (CMR) and fluorodeoxyglucose F-18 positron emission tomography (FDG-PET) have therefore emerged as the principal noninvasive imaging techniques in contemporary practice. CMR provides multiparametric tissue characterization without ionizing radiation, enabling detection of myocardial edema, fibrosis, and perfusion abnormalities, and is widely recommended in guidelines for inflammatory cardiomyopathies [12,13]. FDG-PET, by contrast, assesses regional myocardial glucose uptake and identifies metabolically active inflammation, making it particularly valuable in cardiac sarcoidosis for diagnosis and treatment monitoring [14]. The relative diagnostic performance of these modalities depends on disease phenotype, patient selection, reference standard, and imaging protocol, including CMR sequences and FDG preparation strategies.

Published estimates of diagnostic accuracy for CMR and FDG-PET vary substantially across studies, complicating test selection and interpretation in clinical practice. This meta-analysis therefore aims to generate pooled estimates of sensitivity, specificity, positive predictive value (PPV), and negative predictive value (NPV) for each modality, with secondary reporting of diagnostic odds ratios and summary area under the curve (AUC). We further explore sources of between-study heterogeneity and conduct prespecified subgroup analyses by disease type (myocarditis versus cardiac sarcoidosis), reference standard, and key protocol variables, with the goal of providing pragmatic, clinically relevant guidance.

## Review

Methodology

Study Design and Reporting Framework

This systematic review and meta-analysis evaluated the diagnostic accuracy of CMR imaging and FDG-PET for the assessment of cardiac inflammation. The review was conducted in accordance with the Preferred Reporting Items for Systematic Reviews and Meta-Analyses (PRISMA) 2020 statement for diagnostic accuracy reviews.

Literature Search Strategy

A comprehensive literature search was performed from database inception to the date of final search (October 2025). The following electronic databases were searched: PubMed/MEDLINE, Embase, Scopus, and the Cochrane Library. To minimize publication bias, gray literature sources were also screened, including conference proceedings and abstracts, ClinicalTrials.gov, and preprint servers (medRxiv and bioRxiv). In addition, reference lists of eligible articles and relevant prior reviews were hand-searched. Searches were restricted to studies involving human subjects and published in English. No restrictions were applied based on publication status.

The core search strategy combined controlled vocabulary terms and free-text keywords related to cardiac inflammation and imaging modalities. The general search strategy was as follows and was adapted for all databases based on their specific search pattern: (“myocarditis” OR “cardiac sarcoidosis” OR “inflammatory cardiomyopathy” OR “cardiac inflammation”) AND (“cardiac magnetic resonance” OR “CMR” OR “magnetic resonance imaging”) OR (“FDG-PET” OR “18F-FDG” OR “positron emission tomography”) AND (“diagnostic accuracy” OR “sensitivity” OR “specificity” OR “ROC”).

Eligibility Criteria

Eligible studies included adult populations with suspected or established cardiac inflammation, including myocarditis and cardiac sarcoidosis, in whom CMR or FDG-PET was used as an index diagnostic test and compared against a prespecified reference standard. Acceptable reference standards were defined as the following: endomyocardial biopsy (EMB); guideline-based or well-documented diagnostic criteria; or multidisciplinary expert clinical diagnosis, defined as concordant assessment by at least two experienced clinicians (e.g., cardiologist, electrophysiologist, or cardiac radiologist) integrating clinical data, imaging findings, laboratory testing, and follow-up.

Studies were required to report, or allow reconstruction of, 2×2 diagnostic accuracy data for at least one primary metric (sensitivity, specificity, PPV, or NPV). Eligible designs included prospective and retrospective cohort studies, cross-sectional diagnostic accuracy studies, and systematic reviews with extractable primary data. Pediatric-only cohorts, case reports, editorials, narrative reviews, and abstracts without sufficient data were excluded.

Study Selection and Data Extraction

Titles and abstracts were screened independently by two reviewers, followed by a full-text review of potentially eligible studies. Inter-rater agreement was quantified using Cohen’s kappa at both screening stages. Disagreements were resolved by consensus discussion and, when necessary, adjudication by a third reviewer. Data extraction was performed independently by the same reviewers using a standardized extraction form. Extracted variables included first author, publication year, country, study design, sample size, patient demographics, imaging protocols (including CMR sequences and FDG preparation methods), reference standard, and diagnostic 2×2 contingency data.

Risk of Bias and Applicability Assessment

Risk of bias and concerns regarding applicability were assessed using the Quality Assessment of Diagnostic Accuracy Studies-2 (QUADAS-2) tool across the four domains of patient selection, index test, reference standard, and flow and timing [15]. Study-level judgments (low, high, or unclear risk) were assigned independently by two reviewers, with disagreements resolved by consensus or third-party arbitration.

Statistical Analysis

Quantitative synthesis was conducted using random-effects models to account for anticipated clinical and methodological heterogeneity related to patient populations, imaging protocols, and reference standards. Primary outcomes were pooled sensitivity, specificity, PPV, and NPV. When sufficient data were available, hierarchical summary receiver-operating characteristic (HSROC) modeling was applied to jointly summarize sensitivity and specificity and to assess threshold effects. A minimum of four studies per modality and outcome was prespecified for HSROC modeling to reduce the risk of unstable model convergence. When fewer studies were available, pooled estimates were calculated using random-effects methods and presented with corresponding forest plots.

Between-study heterogeneity was quantified using the I² statistic. Prespecified subgroup analyses examined diagnostic performance according to disease type (myocarditis versus cardiac sarcoidosis), reference standard, and key protocol variables where data permitted. All analyses were performed using Review Manager (RevMan) version 5.4 in conjunction with standard diagnostic meta-analysis packages. Analytic decision rules, inclusion thresholds, and modeling assumptions were prespecified and documented.

Results

**Figure 1:**
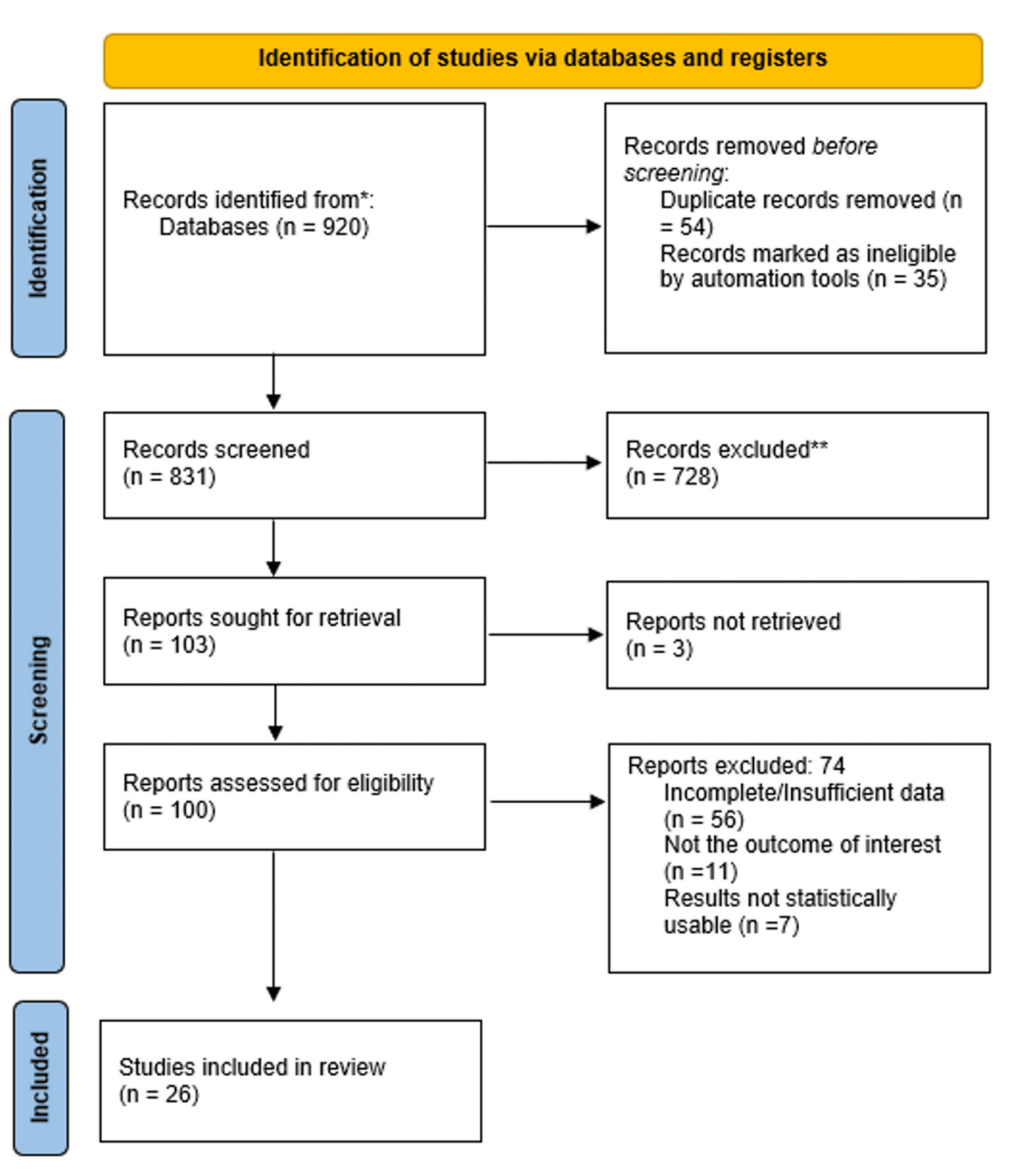
PRISMA Flowchart

A total of 26 studies were included in the final meta-analysis, as seen in the PRISMA flowchart (Figure 1); 17 evaluating CMR and 9 evaluating ^18F-FDG PET (Tables 1-2) [16-41]. All studies enrolled adult patients (≥18 years) with clinically suspected or confirmed inflammatory cardiomyopathy, including myocarditis and cardiac sarcoidosis. Reference standards varied and included endomyocardial biopsy, prespecified clinical diagnostic criteria, or multidisciplinary expert diagnosis. Study-level differences in imaging protocol, patient mix, disease subtype, and methodology produced observable heterogeneity in reported outcomes.

**Table 1:** FDG-PET included studies FDG-PET: fluorodeoxyglucose F-18 positron emission tomography

Authors	Publication Date	Number of Patients	Age Range	Disease Investigated
Nensa et al. [33]	2018	55	26–32	Myocarditis
Peretto et al. [34]	2022	75	33–61	Arrhythmic Myocarditis
Ishimaru et al. [35]	2005	62	38–72	Cardiac Sarcoidosis
Norikane et al. [36]	2017	20	33–79	Cardiac Sarcoidosis
Okumura et al. [37]	2004	22	37–78	Cardiac Sarcoidosis
Blankstein et al. [38]	2014	118	41–63	Cardiac Sarcoidosis
Schildt et al. [39]	2017	231	20–80	Cardiac Sarcoidosis
Osiecki et al. [40]	2020	14	14–79	Cardiac Sarcoidosis
Tahara et al. [41]	2010	24	27–80	Cardiac Sarcoidosis

**Table 2:** CMR included studies CMR: cardiac magnetic resonance

Authors	Publication Date	Disease Investigated	Number of Patients	Age Range
Bohnen et al. [16]	2015	Myocarditis	31	34–63
Darlington et al. [17]	2018	Cardiac sarcoidosis	42	28–78
Abdel-Aty et al. [18]	2005	Acute myocarditis	48	19–61
Mahrholdt et al. [19 ]	2004	Myocarditis	32	30–60
Jeserich et al. [20]	2010	Myocarditis	98	35–70
Laissy et al. [21]	2002	Acute myocarditis	20	29–76
Luetkens et al. [22]	2015	Acute myocarditis	84	22–64
Lurz et al. [23]	2012	Myocarditis	132	39–63
Matsumoto et al. [24]	2015	Cardiac sarcoidosis	17	29–79
Orii et al. [25]	2020	Cardiac sarcoidosis	50	60–75
Patel et al. [26]	2009	Cardiac sarcoidosis	81	35–57
Röttgen et al. [27]	2010	Acute myocarditis	131	21–79
Schwab et al. [28]	2015	Infarct-like myocarditis	78	20–50
Smedema et al. [29]	2005	Cardiac sarcoidosis	58	37-54
Manins et al. [30]	2017	Cardiac sarcoidosis	20	31-56
Kouranos et al. [31]	2017	Cardiac sarcoidosis	321	38-56
Von Knobelsdorff-Brenkenhoff et al. [32]	2017	Acute myocarditis	36	23-38

**Figure 2:**
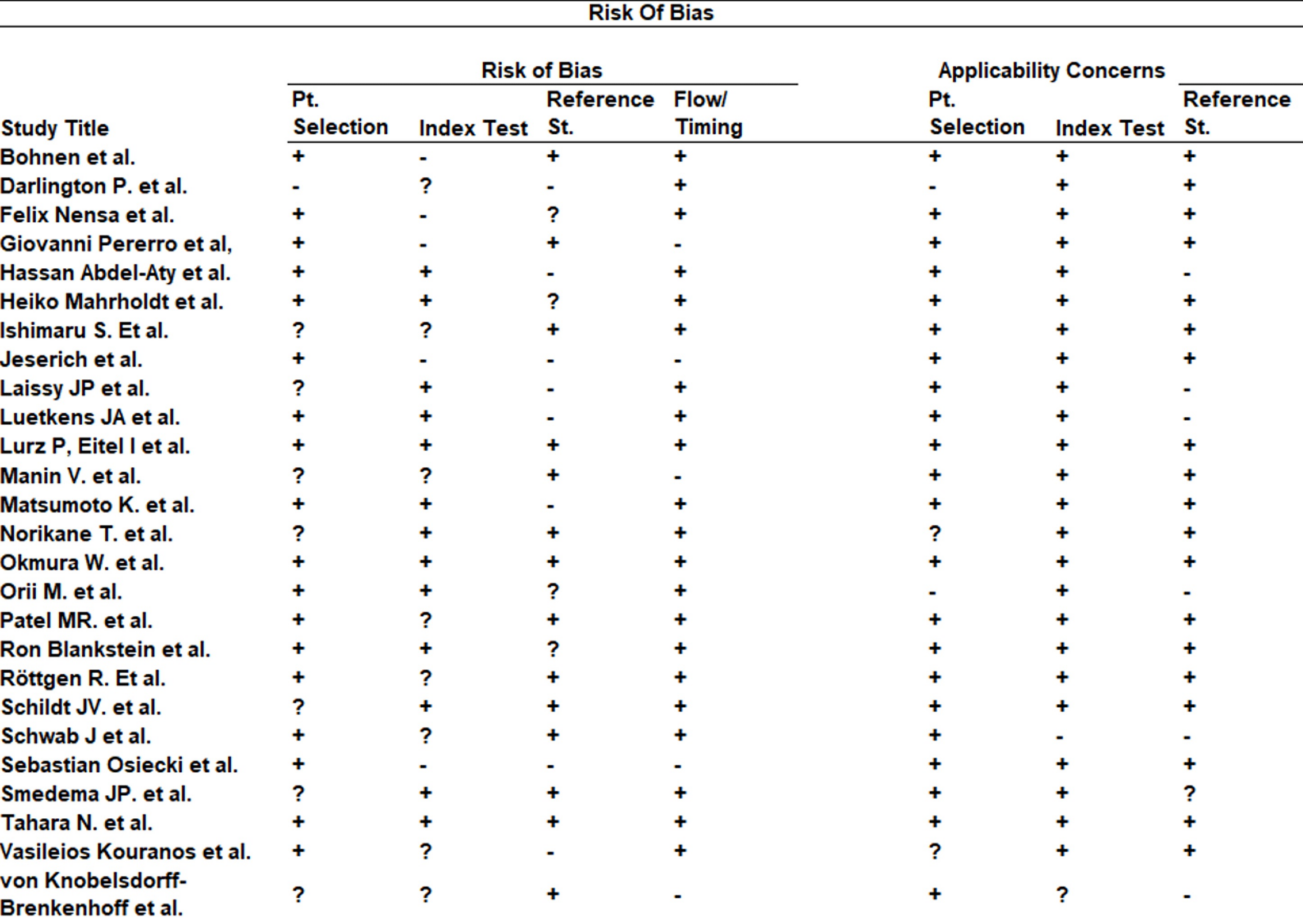
QUADAS 2 risk of bias assessment results References in order [16-41] QUADAS 2: Quality Assessment of Diagnostic Accuracy Studies-2

The Quality Assessment of Diagnostic Accuracy Studies-2 (QUADAS-2) assessment is shown in Figure 2.

**Figure 3:**
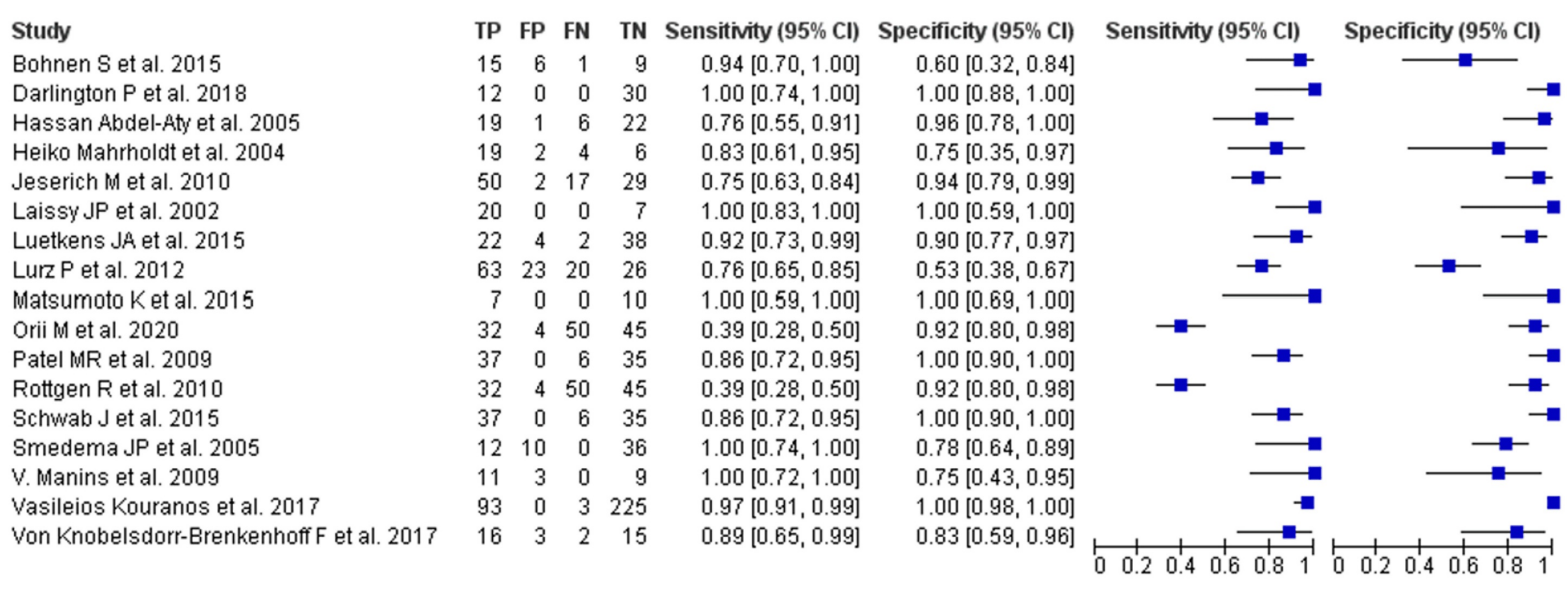
CMR Forest Plots for Sensitivity and Specificity References in order [16-32] CMR: cardiac magnetic resonance

In pooled analyses, CMR demonstrated strong diagnostic performance: pooled sensitivity 0.89 (95% CI 0.85-0.92) and pooled specificity 0.87 (95% CI 0.83-0.90). Heterogeneity was moderate (I² = 41% for sensitivity, I² = 46% for specificity). Study-level sensitivity ranged widely (0.34-1.00) and specificity from 0.60-1.00; studies that used multiparametric CMR protocols (T1/T2 mapping plus late gadolinium enhancement (LGE)) tended to report the highest accuracy. The observed between-study variation for CMR appears mainly attributable to differences in technique, timing relative to disease course (acute versus chronic), interpretation criteria, and the chosen reference standard. Forest plots are shown in Figure 3.

**Figure 4:**
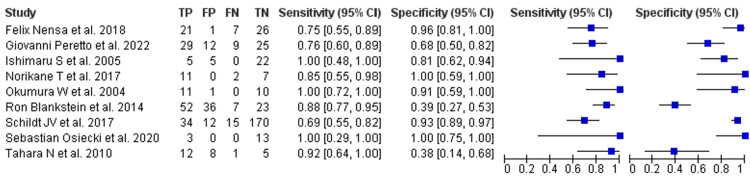
FDG-PET Forest Plots for Sensitivity and Specificity References in order [33-41] FDG-PET: fluorodeoxyglucose F-18 positron emission tomography

FDG-PET showed a pooled sensitivity of 0.93 (95% CI 0.89-0.96) and a pooled specificity of 0.71 (95% CI 0.62-0.79). Heterogeneity was I² = 36% for sensitivity and I² = 48% for specificity. Across studies, sensitivity ranged from 0.69 to 1.00 and specificity from 0.38 to 1.00. The greater dispersion in FDG-PET specificity was predominantly linked to variation in myocardial suppression and dietary preparation: incomplete suppression and nonspecific uptake increased false positives in several reports. When strict suppression protocols were applied, FDG-PET performed especially well in cohorts with metabolically active disease, such as many cardiac sarcoidosis series. Forest plots are shown in Figure 4.

**Figure 5:**
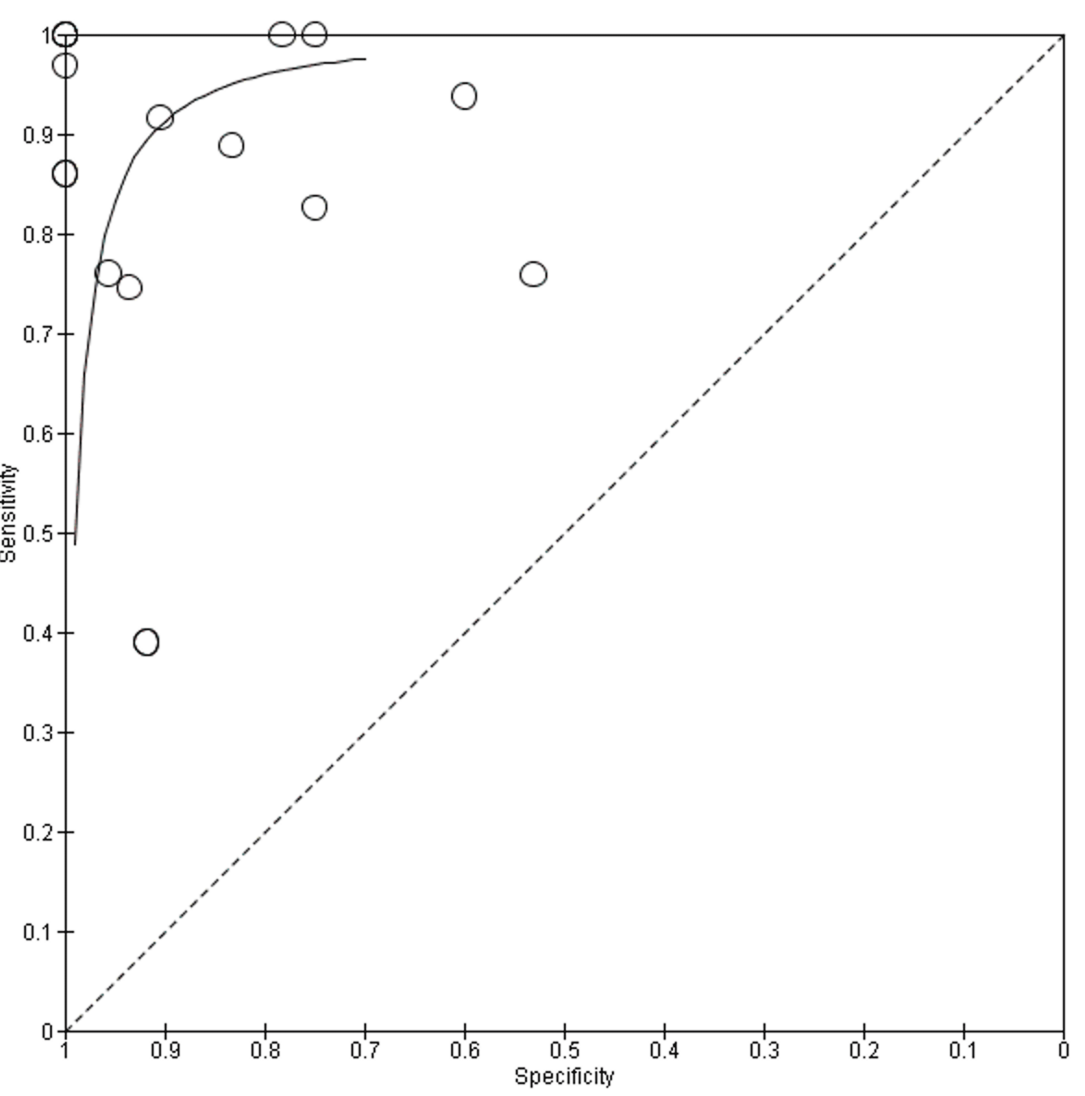
CMR HSROC Curve References in order: [16-32] CMR HSROC: cardiac magnetic resonance hierarchical summary receiver-operating characteristic

**Figure 6:**
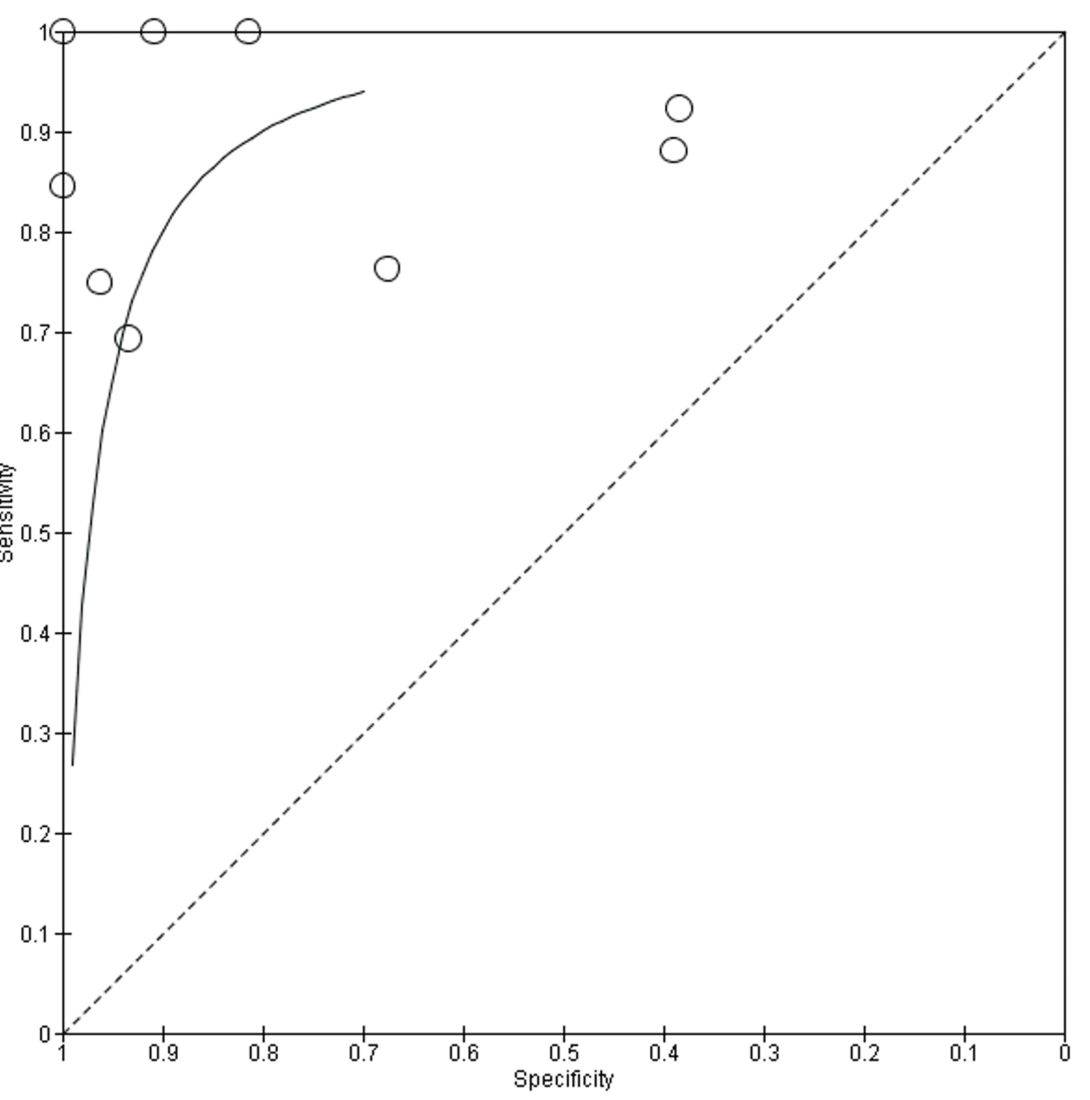
FDG-PET HSROC Curve References in order: [33-41] CMR HSROC: cardiac magnetic resonance hierarchical summary receiver-operating characteristic

The HSROC curve for CMR (Figure 5) shows a strong clustering of data points in the upper-left corner, reflecting consistently high sensitivity and specificity across most studies. The curve itself rises steeply and plateaus near the top, indicating robust diagnostic accuracy. While a few studies show lower specificity or sensitivity, likely due to differences in imaging protocols or disease stage, most fall within a narrow and favorable range. This supports earlier findings that CMR is a reliable tool for detecting key features of myocardial inflammation, including edema and fibrosis, particularly when advanced techniques like T1/T2 mapping and LGE are used. The HSROC curve for FDG-PET (Figure 6) also demonstrates strong diagnostic potential but with a wider spread of data points, especially along the specificity axis. This variation likely reflects the known challenges with myocardial suppression and patient preparation protocols, which can affect tracer uptake and interpretation. Despite this, the curve remains close to the top-left corner, and several studies report near-perfect sensitivity. 

Discussion

This meta-analysis highlights that both CMR and FDG-PET are valuable for diagnosing cardiac inflammation, but each has distinct strengths and limitations in clinical practice. In myocarditis, FDG-PET and CMR findings generally agree well; with a study showing CMR establishing a positive diagnosis with a sensitivity of 67% and a specificity of 91%, though novel T1 mapping techniques have demonstrated higher values of 89% sensitivity and 90% specificity. In comparison, a prospective study evaluating FDG-PET reported a sensitivity of 74% and a notably high specificity of 97% relative to CMR [42]. In another study of myocarditis, FDG-PET showed roughly 89% specificity, supporting FDG-PET’s role when CMR is unsuitable [43]. Importantly, FDG-PET can be clinically useful where CMR is limited (for example, in patients with implanted devices, irregular rhythm, or when artifacts impair MR), making it a complementary tool rather than a direct replacement for CMR [43].

For cardiac sarcoidosis (CS), multiple studies indicate that both modalities have high sensitivity but only moderate specificity when applied across heterogeneous cohorts [44-47]. FDG-PET is especially sensitive for metabolically active granulomatous inflammation, whereas CMR provides detailed tissue characterization and a high NPV in many series [45,46]. Real-world comparative studies also show substantial spatial concordance between PET and LGE-CMR in CS cohorts, though PET may detect inflammatory activity that precedes or occurs without overt scar on CMR [46]. These complementary detection patterns explain why neither modality alone is uniformly “best” across every patient subgroup.

Disease phenotype and timing are major determinants of test performance. Acute, edematous myocarditis is more likely to be detected by T2-weighted/parametric CMR techniques, whereas focal granulomatous inflammation (typical in sarcoidosis) frequently shows FDG positivity even when scarring is modest [42,46]. Our pooled results and prior meta-analyses both demonstrate notable between-study heterogeneity; contributing factors include patient selection (tertiary referral and arrhythmic cohorts inflate pre-test probability), variable reference standards (EMB, guideline-based clinical criteria, or multidisciplinary diagnosis), and evolving imaging criteria (for example, classic vs updated Lake Louise CMR criteria or different PET interpretation schemes) [44,47]. Protocol elements matter: failed myocardial suppression and dietary preparation are nontrivial; in prospective PET/MRI work, failed suppression excluded ~12% of studies, and incomplete suppression increases false positives and lowers specificity [42].

Practical considerations shape modality choice. CMR is widely available, free of ionizing radiation, and provides multiparametric tissue characterization that aids diagnosis, prognostication, and follow-up. FDG-PET demands radiotracer logistics and strict patient preparation (high-fat/low-carbohydrate diets, prolonged fasting, and sometimes heparin), and centers must manage suppression failures and interpret focal versus diffuse uptake carefully. Hybrid PET/MRI systems can combine strengths and offer excellent concordance in selected series, but such platforms are expensive and uncommon; formal position statements and joint guidance note feasibility and potential value while urging larger prospective work to establish incremental benefit and cost-effectiveness [48]. Consequently, many clinicians use CMR as first-line imaging in suspected myocarditis or CS and reserve FDG-PET for equivocal cases, when PET will change management (for example, to guide biopsy, map active inflammation for targeted therapy, or monitor immunosuppression) [43,46].

Imaging also informs treatment and surveillance. FDG-PET identifies metabolically active inflammation and is therefore useful for monitoring response to immunosuppression and guiding decisions about tapering therapy. CMR parameters (T2 maps, LGE burden) offer structural and prognostic information and can show remodeling after therapy. Small longitudinal series show that PET activity often declines earlier than LGE changes, indicating complementary roles: PET for inflammatory activity, CMR for structural injury, and longer-term scar [42,46].

From a patient perspective, cost and access substantially influence the choice between CMR and FDG-PET when evaluating suspected myocardial inflammation. In the United States, advanced cardiac imaging frequently entails out-of-pocket expenses despite insurance coverage. Medicare and many private insurers cover diagnostic imaging but require deductibles and coinsurance for outpatient studies, often leaving patients responsible for several hundred dollars per examination, while uninsured patients may face the full billed amount. In contrast, most Western European healthcare systems provide medically indicated CMR and FDG-PET with minimal or no direct patient payment, shifting financial considerations away from the individual patient and toward system-level resource allocation [49,50].

Geographic availability further shapes patient experience. Cardiac FDG-PET remains concentrated in tertiary or academic centers, and many US regions lack local access, requiring patients to travel long distances and incur indirect costs such as transportation and time away from work. Cardiac MRI is more broadly available at regional hospitals and imaging centers, reducing travel burden for many patients. Similar patterns are observed in Europe, where PET access is centralized, but MRI availability is widespread within public hospital networks. These access differences frequently determine which modality is pursued first in real-world practice [51].

Patient preparation and time commitment also differ meaningfully between modalities. CMR typically requires minimal preparation and is completed within a single visit, with scan times of approximately 30-60 minutes. FDG-PET, by contrast, requires strict dietary preparation; often a low-carbohydrate, high-fat diet for at least 24 hours, along with fasting and a prolonged appointment on the day of imaging to allow for tracer uptake. These requirements increase patient burden and contribute to nondiagnostic studies when myocardial glucose suppression is inadequate [52,53].

## Conclusions

This analysis is limited by heterogeneity in the source literature, including differences in study design, patient selection, imaging protocols, and reference standards. Many studies were single-center or retrospective with highly selected populations, limiting generalizability. Some relied on composite clinical criteria rather than histologic confirmation, and endomyocardial biopsy, when performed, remains susceptible to sampling error in patchy disease. Publication bias cannot be excluded, and the absence of patient-level data restricted detailed subgroup analyses. Emerging imaging techniques and increasing use of PET/MR may also influence diagnostic performance beyond the period studied.

Clinically, these findings reinforce current diagnostic strategies. CMR remains the preferred first-line noninvasive test for suspected myocarditis and many cases of CS due to its consistent specificity and ability to characterize myocardial tissue. FDG-PET is most informative when active inflammation is suspected or when CMR findings are inconclusive, provided strict patient preparation is applied. Future progress will require multicenter prospective studies comparing CMR, PET, and PET/MR using standardized protocols and patient-level data. In the meantime, imaging results should be interpreted in the broader clinical context, considering pre-test probability, disease chronicity, and technical factors.
